# Evidence for Fibroblast Growth Factor-2 as a Mediator of Amphetamine-Enhanced Motor Improvement following Stroke

**DOI:** 10.1371/journal.pone.0108031

**Published:** 2014-09-17

**Authors:** William A. Wolf, Jody L. Martin, Gwendolyn L. Kartje, Robert G. Farrer

**Affiliations:** 1 Research Service, Edward Hines, Jr. Veterans Administration Hospital, Hines, Illinois, United States of America; 2 Neurology Service, Edward Hines, Jr. Veterans Administration Hospital, Hines, Illinois, United States of America; 3 Department of Cell and Molecular Physiology, Loyola University, Maywood, Illinois, United States of America; 4 Department of Molecular Pharmacology and Therapeutics, Loyola University, Maywood, Illinois, United States of America; 5 Department of Anatomy and Cell Biology, University of Illinois at Chicago, Chicago, Illinois, United States of America; Massachusetts General Hospital/Harvard Medical School, United States of America

## Abstract

Previously we have shown that addition of amphetamine to physical therapy results in enhanced motor improvement following stroke in rats, which was associated with the formation of new motor pathways from cortical projection neurons of the contralesional cortex. It is unclear what mechanisms are involved, but amphetamine is known to induce the neuronal release of catecholamines as well as upregulate fibroblast growth factor-2 (FGF-2) expression in the brain. Since FGF-2 has been widely documented to stimulate neurite outgrowth, the present studies were undertaken to provide evidence for FGF-2 as a neurobiological mechanism underlying amphetamine-induced neuroplasticity. In the present study rats that received amphetamine plus physical therapy following permanent middle cerebral artery occlusion exhibited significantly greater motor improvement over animals receiving physical therapy alone. Amphetamine plus physical therapy also significantly increased the number of FGF-2 expressing pyramidal neurons of the contralesional cortex at 2 weeks post-stroke and resulted in significant axonal outgrowth from these neurons at 8 weeks post-stroke. Since amphetamine is a known releaser of norepinephrine, *in vitro* analyses focused on whether noradrenergic stimulation could lead to neurite outgrowth in a manner requiring FGF-2 activity. Primary cortical neurons did not respond to direct stimulation by norepinephrine or amphetamine with increased neurite outgrowth. However, conditioned media from astrocytes exposed to norepinephrine or isoproterenol (a beta adrenergic agonist) significantly increased neurite outgrowth when applied to neuronal cultures. Adrenergic agonists also upregulated FGF-2 expression in astrocytes. Pharmacological analysis indicated that beta receptors and alpha1, but not alpha2, receptors were involved in both effects. Antibody neutralization studies demonstrated that FGF-2 was a critical contributor to neurite outgrowth induced by astrocyte-conditioned media. Taken together the present results suggest that noradrenergic activation, when combined with physical therapy, can improve motor recovery following ischemic damage by stimulating the formation of new neural pathways in an FGF-2-dependent manner.

## Introduction

Stroke remains a leading cause of death and disability worldwide [1]. In many cases some spontaneous functional recovery occurs, but this is rarely complete and patients continue to suffer from sensorimotor, cognitive or other neurologic impairments. It is estimated that 50% of patients are left with motor disability that predominantly occurs in the upper limbs [2], [3]. Neural plasticity, defined as the functional reorganization of the brain, occurs following ischemic injury and can involve regions quite distant from the lesion as well as peri-lesional areas [4], [5]. Physical therapy has been the mainstay of rehabilitative strategies for improved recovery of motor function following brain injury. However, there is considerable interest in further improving outcome through the use of adjunct medication.

In general, drugs that promote neural plasticity appear to facilitate physical therapy-aided motor improvement although there is great variability in outcomes depending on study design, drugs and rehabilitation methods employed. Drugs that increase the synaptic activity of the monoamines norepinephrine (NE), dopamine (DA) and serotonin(5-HT), alter gene transcription, protein synthesis and dendritic outgrowth in a manner similar to what is seen in animals exposed to an enriched environment and/or exercise and which appears to be associated with improved functional outcome following brain injury [6]–[12]. In particular, drugs that enhance central noradrenergic activity, such as amphetamine (which induces the neuronal release of predominantly NE and DA), have been the most widely studied drugs for improving motor function following stroke [13]–[20]. Although amphetamine has shown great promise in pre-clinical studies it has produced mixed resulted in clinical trials [14], [17], [19], [21], [22]. The variability in clinical efficacy combined with the tendency of amphetamine to increase mortality due to cardiovascular side effects have hindered its development as a rehabilitation adjunct in stroke [17], [22]. A greater understanding of the salient mechanisms underlying amphetamine-enhanced motor improvement following stroke would facilitate the development of safer, more effective therapies. To this end, considerable evidence suggests a role for fibroblast growth factor-2 (FGF-2) in mediating motor improvement following amphetamine or related drugs.

Preclinical studies indicate that fibroblast growth factor-2 (FGF-2; also known as basic fibroblast growth factor) FGF-2 is one of a number of neurotrophic factors that are upregulated in response to brain injury presumably to subserve protective/restorative roles and to restore homeostasis [23]–[26]. The cellular actions of FGF-2 include the promotion of cellular proliferation, differentiation and migration, as well as induction of neuronal fiber outgrowth. Central administration of FGF-2 within 24 hr of experimental stroke has been shown to improve motor recovery and upregulate growth-associated protein 43, a marker of axonal growth [27], [28]. Conversely, central application of neutralizing antibodies to FGF-2 have been shown to impair motor recovery following cortical damage [29]. The possibility that FGF-2 is involved in amphetamine-related neural plasticity is supported by several observations. First, short-term amphetamine (3 injections, once every other day) induces a persistent increase in FGF-2 in the brain that lasts for at least 1 month [30], [31]. This short term regimen is similar to the drug treatment used in our previous study in which we demonstrated an amphetamine-mediated improvement in motor function following stroke that was associated with new neuronal projections originating from the contralesional cortex [18]. Second, central administration of a neutralizing antibody to FGF-2 blocks the persistent plasticity-related behavioral/motor changes (referred to as “sensitization”) caused by short, intermittent regimens of amphetamine [31]. Taken together the available evidence led us to investigate whether FGF-2 is an important contributor to the rehabilitative potential of amphetamine. The present results support the hypothesis that enhanced noradrenergic activity induced by amphetamine upregulates FGF-2, which leads to enhanced axonal outgrowth and improved motor function following stroke.

## Materials and Methods

### Animals

Adult male Long Evans, black-hooded rats (250–300 g) were used. All experimental procedures were in accordance with the *Guide for the Care and Use of Laboratory Animals* and approved by the Institutional Animal Care and Use Committee at Hines VA and the Animal Care Committee at University of Illinois at Chicago. Animals were maintained in a temperature and humidity controlled room under a 12∶12-h light/dark cycle. Food intake was moderately restricted throughout the study to maintain body weight at 95% of ad libitum weight. Water was available ad libitum.

### Initial training and stroke surgery

Upon arrival to the vivarium animals were randomly assigned to groups that would ultimately comprise the experimental cohorts. This was done in order to reduce aggression caused by future shuffling of animals among established groups. Prior to stroke surgery animals were trained to criterion on the skilled forelimb reaching task and assessed for their baseline performance on the ladder rung walk test. The pre-operative criterion for skilled forelimb reaching was at least 16 successes in 20 attempts for 3 consecutive days. Animals then underwent middle cerebral artery occlusion essentially as previously described [18]. Briefly, rats were anesthetized with ketamine/xylazine (75 mg/kg ketamine plus 5 mg/kg xylazine, i.p.). Then a vertical 2 cm long incision was made between the eye and ear, and the temporalis muscle was retracted. A burr hole was made to expose the MCA and it was permanently occluded with a 10-0 suture. The CCA ipsilateral to the MCA was temporarily occluded for 45 min with a micro vessel clamp. The wounds were then closed and animals were allowed to recover. Experimental assignment of each cohort was done on a random basis (i.e. not based on any post-stroke performance).

### Housing/Physical therapy

Immediately after surgery, groups of rats were randomly allocated to different experimental conditions as follows:

1. vehicle administration/control housing (VEH+CON)
2. vehicle administration/rehabilitation (VEH+REHAB)
3. amphetamine administration/rehabilitation (AMPH+REHAB).

Amphetamine administration in the absence of physical therapy was omitted as our previous study demonstrated that amphetamine alone does not improve motor performance or enhance neurite outgrowth from contralesional cortex [18]. Control housing conditions (CON) consisted of singly housed animals in a standard Plexiglas cage (24 cm×36 cm×15 cm) with no additions. Rehabilitation (REHAB) consisted of housing animals in an enriched environment with supplementary sessions of focused activity. The enriched environment consisted of group-housed animals (5–6 per cohort) in multi-level “condos,” which were 32″ wide×32″ deep×60″ tall, constructed of Plexiglas on 3 sides with air holes throughout and fitted with hinged screen doors of 1 cm square mesh and a mesh top. Each condo was furnished with inclined ladders, hanging toys, climbing cylinders, chewable material, and igloos. Once a week novel objects were introduced. Focused activity sessions (20 min duration) were performed as previously described and consisted of actively placing animals on climbing apparatuses that relied heavily on the use of forelimbs, but were distinct from the specific tasks being assessed (climbing cylindrical grid, sisal covered vertical pole, inclined ladder and ramps) [18], [20].

### Drug treatment

On days of drug administration (Post-op Days 2, 5 and 8) activity sessions began after completion of daily behavioral testing and fifteen minutes following drug or vehicle administration. D-amphetamine sulfate (2 mg/kg based on salt wt; Sigma Chemical Co., St. Louis, MO, USA) or vehicle (0.9% sterile saline) was administered subcutaneously.

### Behavioral tests

Skilled forelimb reaching was assessed by counting the number of times an animal grasped a sucrose pellet on the first attempt and placed it into the mouth (i.e. “first reach success”). Each testing session consisted of 20 reaching opportunities using the preferred forelimb. Attempts using the non-preferred forelimb were not included in analyses. Tests were performed daily. Ladder rung walk utilized a horizontal ladder runway (1 m×10 cm) with 2 Plexiglas walls on either side. Testing sessions consisted of 3 runway crossings and were performed weekly. Forelimb foot errors were defined as either a complete miss or complete slip. Details of behavioral testing can be found in our previous studies [18], [20]. Investigators performing behavioral assessments were blind to the treatment group.

### Neuroanatomical tracing

After 8 weeks of behavioral testing, animals were anesthetized with ketamine/xylazine (75 mg/kg ketamine plus 5 mg/kg xylazine, i.p.). The sensorimotor cortex opposite to the stroke lesion site was exposed, and 2 injections of 1 µl each of a 10% biotinylated dextran amine (BDA) solution (10,000 MW, Molecular Probes/Life Technologies, Grand Island, NY, USA) were placed stereotaxically into the forelimb sensorimotor cortex (0.5 mm anterior, 2.5 mm lateral, 1.5 mm depth, relative to bregma). Two weeks after BDA injection, animals were overdosed with sodium pentobarbital (100 mg/kg; i.p.) and perfused transcardially with 4% paraformaldehyde. Brains and spinal cords were removed, placed in 30% sucrose for 1–2 days, embedded in Tissue-Tek OCT compound (Sakura Finetek, USA, Torrance, CA, USA), frozen, and stored at −80°C. Alternate coronal cryosections (30 µm thick) were reacted for BDA positive fibers or processed with Nissl stain and analyzed for lesion location and extent as previously described [18].

### Neuroanatomical analysis of fiber crossing

Anatomical structures were identified with the atlas of Paxinos and Watson. Quantification of sprouting corticorubral fibers from the contralesional side to the deafferented (ipsilesional) red nucleus was performed by counting all BDA-positive fibers crossing the midline at the level of the red nucleus. The number of labeled cortico-efferent fibers in the cerebral peduncle ipsilateral to the BDA injection site was determined using NIH Image and used to correct for inter-animal variances in BDA tracing as described previously [18]. For all analyses the slides were coded and investigators were blind to the treatment group.

### Immunohistochemistry for FGF-2

Following pentobarbital overdose animals were perfused with phosphate-buffered saline (PBS) followed by 4% paraformaldehyde in 0.1 M PBS. Animals were sacrificed at either 2 weeks post-op or 8 weeks post-op. Brains were post-fixed for 1 hr, cryoprotected then frozen. Free-floating cryostat sections (30 µm) were incubated in PBS containing 0.1% Triton X-100 (PBST) containing 5% normal goat serum and primary antibody (1∶400 ms×FGF-2; clone 3; BD Biosciences, San Jose, CA, USA) for 48 hr at 4°C. After PBST washes, sections were incubated in goat anti-mouse (1∶100; Covance Inc, Dedham, MA, USA) for 1 hr at room temperature, washed and then incubated with mouse ClonoPAP (1∶250; Covance Inc, Dedham, MA, USA) for 1 hr at room temperature. Visualization was performed using diaminobenzidine (ImmPACT DAB, Vector Labs, Burlingame, CA, USA). Serially adjacent sections were Nissl-stained using toluidine blue for total cell counts and assessment of lesion size. For all analyses the slides were coded and investigators were blind to the treatment group.

### Stereologic analysis of FGF-2-labeled cells

A systematic random sampling procedure was followed using every 10^th^ section of the brain from approximately +2.0 mm to −1.0 mm from bregma. Counting was restricted to the region of the motor cortex extending from the primary motor cortex (medial extent of counting region) to the primary somatosensory forelimb area (lateral extent of counting region). After outlining the region of interest cell counting was performed following the optical dissector protocol (Stereologer; Stereology Resource Center, St. Petersberg, FL, USA). Section thickness following histological processing averaged approximately 15 um. The guard zone and optical dissector height were set at 2 um and 10 um, respectively. Total cell population = total cells counted×1/*ssf*×1/*asf*×1/*hsf*. A Gunderson coefficient of error of estimation <0.1 was obtained.

### Stroke size analysis and exclusion criteria

If the lesion did not impinge on the forelimb region of the sensorimotor cortex, and/or if subcortical damage was observed, then the animal was excluded from the study. Stroke volume was quantitatively analyzed on Nissl stained sections using NIH image as described previously by investigators blind to the treatment group [18].

### Primary cell culture

Cell culture media, serum, and antibiotics were obtained from Life Technologies (Grand Island, NY, USA). Primary cultures of rat cortical astrocytes were prepared from postnatal day 2 rat pups essentially as described by McCarthy and de Vellis [32]. Cells were grown and maintained in DMEM supplemented with 10% FBS and 0.25% gentamicin until use. Cells were grown at 37°C in a water-saturated environment in 5% CO2 and passaged twice before use. Prior to use flasks were shaken on an orbital shaker overnight to remove progenitor cells. Immunocytochemical staining for glial fibrillary acidic protein (GFAP) and S100 beta indicated that cell populations were >95% astrocytes.

Primary cultures of rat cortical neurons were obtained from embryonic day 18 cortices obtained from BrainBits (Springfield, IL, USA) prepared per the supplier's instructions. Cells were seeded on 12 mm poly-lysine-coated coverslips (approximately 5×10^3^ neurons/cm2) initially in Neurobasal containing B27 and 0.5 mM glutamax supplemented with 10% FBS. The next day the media was exchanged for fresh Neurobasal containing B27/glutamax in which the FBS was omitted (hereafter referred to as N/B27). After another 24 hr, neurons were placed in fresh N/B27 or astrocyte-conditioned media prepared as described below. Following 24 hr incubation under experimental conditions coverslips were taken for immunocytochemical staining for microtubule associated protein 2 (MAP-2) as described below. Cells were maintained at all times at 37°C in a water-saturated environment with 5% CO2

### Preparation of astrocyte-conditioned media (ACM)

Conditioned media was prepared by exchanging normal growth media with N/B27 containing vehicle or the indicated drugs. Routinely, 20 ml of N/B27 was used for T75 flasks of astrocytes (approximately 10×10^6^ cells). After 6 hr of incubation the media was collected and concentrated 10-fold using a 9000 MW cut-off centrifugal concentrator. This concentrate was re-constituted by 10 fold dilution with fresh N/B27 immediately prior to application to neurons.

### Immunocytochemistry

Coverslips with neurons were fixed with 4% paraformaldehyde/4% sucrose on ice, washed, permeabilized with 0.1% triton X-100 for 5 min at room temperature, washed, blocked for 30 min at room temperature with 5% BSA/5% normal donkey serum and then incubated overnight at 4°C with primary antibody (rabbit anti-MAP-2 at 1∶500; EMD Millipore, Billerica, MA, USA). Coverslips were then washed, incubated with secondary antibody (Cy2-conjugated donkey anti-rabbit; Jackson ImmunoResearch, West Grove, PA, USA) for 1 hr at room temperature, washed and incubated with DAPI and mounted on slides with Fluoromount-G (Southern Biotech, Birmingham, AL, USA).

### Immunoblotting

Following the indicated treatments astrocyte whole cell lysates were prepared in NuPage LDS sample buffer (Life Technologies) supplemented with protease inhibitor cocktail III (EMD Millipore/Calbiochem; final concentrations 1 mM AEBSF, 0.8 uM aprotinin, 50 uM bestatin, 15 uM E-64, 20 uM leupeptin, 10 uM pepstatin A), phosphatase inhibitor cocktails I and II (EMD Millipore/Calbiochem; final concentrations 25 uM p-bromotetramisole, 5 uM cantharidin, 5 nM microcystin LR, 2 mM imidazole, 1 mM NaF, 1.15 mM sodium molybdate, 1 mM sodium orthovanadate, 4 mM sodium tartrate) and 100 mM DTT with heating at 70°C for 10 min. Samples were fractionated on 10% Bis-Tris NuPage gels (Life Technologies) and proteins electrophoretically transferred to PVDF membrane. Following blocking for 90 min at room temperature with 5% BSA in TTBS (25 mM Tris-HCl, pH 7.5 containing 0.1% Tween-20) membranes were incubated in primary antibody (1∶2000; ms×bFGF; clone 3; BD Biosciences, San Jose, CA, USA) overnight at 4°C. Following washes and secondary antibody (1∶6000; goat anti-mouse AP-conjugate; Life Technologies) incubation for 60 min at room temperature the immunoreactive bands were detected by chemiluminescence with ImmunStar AP substrate (BioRad, Hercules, CA, USA) and quantified on a Fluorchem SP imager (Alpha Innotech). Equal loading and uniformity of protein transfer were verified by stripping and reprobing for actin and GAPDH.

### Neurite analysis

Assessment of neurite formation was carried out using NIH Image for PC (ImageJ) on fluorescence photomicrographs. For each experimental condition (i.e. coverslip) micrographs of 4 fields under a 20× objective (approximately 0.3 mm^2^ each) were taken. After counting MAP-2 immunoreactive cells, the total neurite length was obtained by first occluding cell bodies and tracing over neuritic processes in Adobe Photoshop. The image was then inverted and converted to grayscale for quantification using ImageJ. For all analyses investigators were blind to the treatment group. Data are expressed and analyzed as percent of corresponding vehicle control.

## Results

### Amphetamine-enhanced motor performance following stroke is associated with an increase in axonal sprouting from corticomotor projection neurons of the contralesional cortex

**Figure 1:**
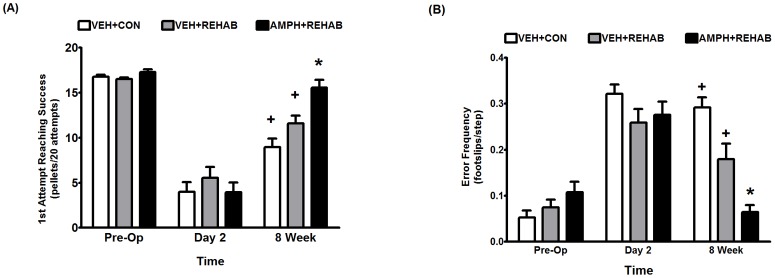
Short term AMPH plus physical rehabilitation improves long term motor performance following stroke. (A) End point analysis of skilled forelimb reaching – All animals enrolled in the study achieved the preoperative criteria of an average of at least 16 successes in 20 attempts for 3 days prior to surgery. At Day 2 Post-op, prior to any treatment, the mean deficit in reaching was not significantly different among treatment groups. Treatment consisted of single housing in standard caging with vehicle (0.9% saline) administered on Days 2, 5 and 8 post-MCAO (VEH+CON), enriched environment with daily sessions of focused activity and vehicle administered on Days 2, 5 and 8 post-MCAO (VEH+REHAB) or enriched environment with daily sessions of focused activity and D-amphetamine sulfate (2 mg/kg based on salt weight) administered on Days 2, 5 and 8 post-MCAO (AMPH+REHAB). A two-way repeated measures ANOVA followed by Holm-Sidak comparisons indicated that reaching performance in AMPH+REHAB was significantly better than VEH+REHAB at eight weeks following MCAO (*, p<0.05). Comparisons also indicated that all groups except AMPH+REHAB still displayed significant deficits in reaching when compared to pre-operative performance (+, p<0.001), which indicates that only AMPH+REHAB induced a recovery to baseline performance in the forelimb reaching task. Behavioral data represent the mean ± SEM for 12 animals/group. (B) End point analysis of ladder rung walking – At Day 2 Post-op, prior to any treatment, the mean deficit in skilled forelimb placement was not significantly different among groups. A two-way repeated measures ANOVA followed by Holm-Sidak comparisons indicated that forelimb placement performance in the ladder rung walk was significantly different among all three groups at eight weeks following MCAO indicating that AMPH+REHAB was significantly better than VEH+REHAB (*, p<0.05). Comparisons also indicated that all groups except AMPH+REHAB still displayed significant deficits in forelimb placement performance at 8 weeks when compared to pre-operative performance (+, p<0.001), which indicates that only AMPH+REHAB induced a recovery to baseline performance in the ladder rung walk. Behavioral data represent the mean ± SEM for 12 animals/group.

Table 1 describes the experimental groups under study and shows that there was no significant difference in infarct volume among these treatment groups. Fig. 1 shows that the addition of amphetamine treatment to physical therapy (i.e. daily sessions of focused activity) significantly enhanced long-term motor improvement following stroke in rats over physical therapy alone. At study end point (8 weeks post-stroke) performance in the skilled forelimb reaching task (Fig. 1-A) and ladder rung walk (Fig. 1-B) were significantly better in animals that received amphetamine plus physical therapy (AMPH+REHAB) than animals that received physical therapy alone (VEH+REHAB). In fact, two-way analysis of variance indicated that only AMPH+REHAB animals recovered to levels of performance that were similar to pre-stroke values.

**Table 1:** Lesion Analysis among Treatment Groups.

Group (n)	Drug/Housing+Activity conditions	Stroke Volume (% of contralesional hemisphere volume)
VEH+CON	Vehicle-treated animals singly	10.1±0.8
(12)	housed under control conditions -	
	no activity sessions	
VEH+REHAB	Vehicle-treated animals group-	11.2±1.8
(12)	housed in enriched environment	
	plus focused activity sessions	
AMPH+REHAB	AMPH-treated animals group-	10.0±1.1
(12)	housed in enriched environment	
	plus focused activity sessions	

Following training animals were subjected to MCAO and distributed among the different treatment groups depicted above. Details of drug treatment, housing and activity conditions are described in more detail in Methods. After 8 weeks of behavioral testing 5 animals were sacrificed for FGF-2 and histological analysis and 7 animals were microinjected with biotinylated dextran amine and sacrificed two weeks later for fiber staining and histological analysis. No significant difference in lesion size among groups was observed (one-way ANOVA). Data represent the mean ± SEM of the indicated number of animals per group.

**Figure 2:**
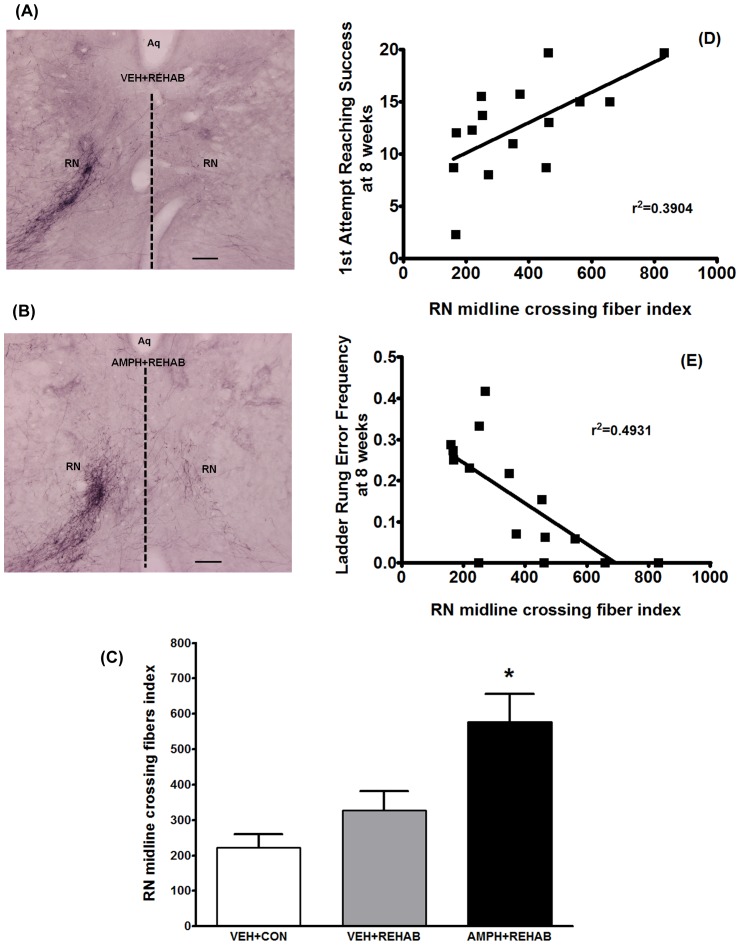
Short term AMPH plus physical rehabilitation enhances axonal outgrowth following stroke. (A) Representative photomicrograph of BDA-positive fiber staining at the level of the red nucleus (RN) in an animal receiving vehicle plus enriched environment with daily sessions of focused activity (VEH+REHAB). Few BDA-positive fibers can be seen crossing the midline (dotted line) from the non-denervated RN (left side) to the denervated side (right). Aq = aqueduct; scale bar represents 100 um. (B) Representative BDA-positive fiber staining at the same level of RN as in (A), but in an animal that received D-amphetamine sulfate (2 mg/kg based on salt weight) plus enriched environment with daily sessions of focused activity (AMPH+REHAB). Note an increase in the number of BDA-positive fibers crossing the midline to the denervated side. Aq = aqueduct; scale bar represents 100 um. (C) Quantification of midline crossing fibers in the area of the red nucleus normalized to the total labeled cerebral peduncle fibers (to correct for differences in the tracing). A one-way ANOVA followed by Student-Newman-Keuls post hoc comparison indicated that midline fiber crossing at the level of the red nucleus was significantly greater following AMPH+REHAB than all other groups (*, p<0.05). Midline fiber crossing data represent the mean ± SEM of a subset of 5 animals/group. Panels (D) and (E) depict linear regression and correlation analyses between midline fiber crossing and performance in skilled forelimb reaching (Panel D) and ladder rung walk (Panel E). A significant positive correlation was found between midline fiber crossing and skilled forelimb reaching (*r* ^2^ = 0.3904, Pearson's *r* = 0.6248; p<0.05). A significant negative correlation was found between midline fiber crossing and footslips (error frequency) in the ladder rung walk (*r* ^2^ = 0.4931, Pearson's *r* = −0.7022; p<0.005).

Anterograde tracing of contralesional corticomotor pathways revealed that amphetamine plus physical therapy also resulted in a significant increase in the number of labeled axons originating from the contralesional cortex and crossing the midline to innervate the deafferentated red nucleus (Fig. 2A–E). Fig. 2-A shows the typical pattern of contralesional corticorubral innervation (RN on left side) in an animal that received physical therapy alone (VEH+REHAB) with few fibers crossing the midline to innervate the deafferentated red nucleus (RN on right side). By comparison Fig. 2-B shows that there is greater innervation of the deafferentated red nucleus (RN on right side) in an animal receiving amphetamine plus physical therapy (AMPH+REHAB). A quantitative summary of these results is shown in Fig. 2-C, in which significantly greater fiber crossing was observed in the group of animals that received AMPH+REHAB as compared to all other groups (p<0.05; ANOVA). Figs. 2-D and E show that the extent of fiber crossing at the level of the red nucleus correlated significantly with improved performance in forelimb reaching (Pearson's *r* = 0.6248; p<0.05) and ladder rung walking (Pearson's *r* = −0.7022; p<0.005) assessed at 8 weeks after stroke.

### Amphetamine-enhanced motor performance following stroke is associated with a short-term increase in FGF-2-expressing pyramidal cells in the contralesional cortex

**Figure 3:**
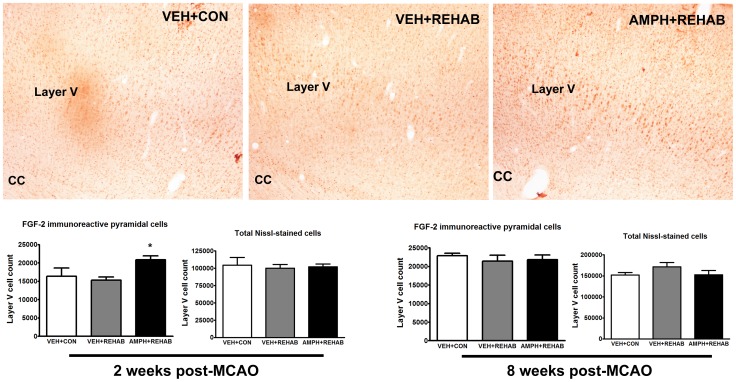
Short term AMPH plus physical rehabilitation increases FGF-2 expression in Layer V pyramidal cells of unlesioned motor cortex. Top panel consists of representative photomicrographs of FGF-2 immunoreactivity in the unlesioned motor cortex. Layer V pyramidal cells are clearly evident and appear more intensely stained in animals receiving AMPH+REHAB as compared to other groups. **Left side**- Stereological assessment of FGF-2-like immunoreactive layer V pyramidal cells and total Nissl-stained cells in unlesioned motor cortex at 2 weeks after MCAO. Animals underwent behavioral procedures and MCAO as described in Methods and Fig. 1, but were sacrificed 2 weeks post-MCAO for histological procedures. One way ANOVA followed by Student-Newman-Keuls comparisons indicated a significant increase in the number of FGF-2-like immunoreactive pyramidal cells in Layer V as compared to all other groups (p<0.05). Total number of Nissl stained cells did not differ among groups. Data represent the mean ± SEM of 6 animals/group. **Right side**- Stereological assessment of FGF-2-like immunoreactive layer V pyramidal cells and total Nissl-stained cells in unlesioned motor cortex at 8 weeks after MCAO. A subset of animals that contributed to the behavioral data in Fig. 1 were used for assessment of FGF-2 expression. Data represent the mean ± SEM of 7 animals/group.

Since amphetamine has been reported to upregulate growth factors in the brain, such as FGF-2, that could lead to axonal growth we assessed the effects of amphetamine on cellular FGF-2 expression [30], [33]–[35]. Subsets of animals were sacrificed at 2 weeks post-stroke and 8 weeks post-stroke (end-point) for stereological assessment of FGF-2 expressing cells in the contralesional cortex, which represents the origin of new corticomotor pathways (Fig. 3). At 2 weeks post-stroke, animals that received amphetamine plus physical therapy (AMPH+REHAB) exhibited a significant increase of approximately 40% in FGF-2-expressing Layer V cortical projection neurons in the unlesioned sensorimotor cortex over animals receiving physical therapy alone (VEH+REHAB; Fig. 3, p<0.05). This effect was not apparent at 8 weeks post-stroke. Total cell number in Layer V, as indicated by Nissl-stained cells, was not different between groups at either time point.

### Neither amphetamine nor norepinephrine directly stimulate neurite outgrowth in cultured primary neurons

**Figure 4:**
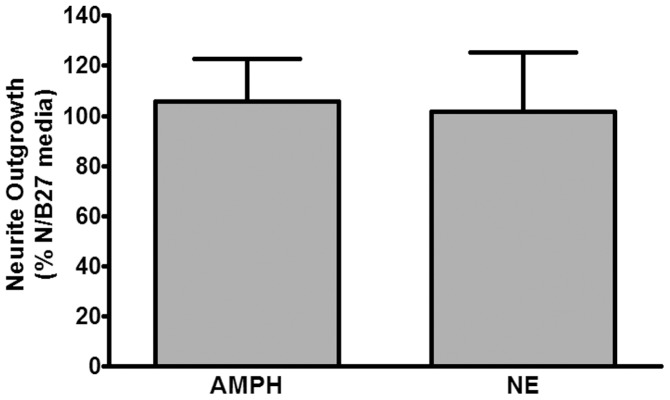
Direct treatment of primary cortical neurons with amphetamine or norepinephrine for 24 hr has no effect on neurite outgrowth. Cortices from E18 rats were dissociated and plated on 12 mm poly-lysine-coated coverslips and allowed to equilibrate as described in Methods. Amphetamine (10 uM), norepinephrine (10 uM) or vehicle was added and incubated with neurons for 24 hr. Neurons on coverslips were fixed and stained for MAP2 and analyzed for neurite outgrowth as described in Methods. Data represent the mean ± SEM of 5 independent observations.

Although upregulation of FGF-2 by amphetamine has previously been characterized as being localized to astrocytes the data from Fig. 3 suggested a potential upregulation in neurons [33]. Since the primary pharmacological action of amphetamine is to induce the neuronal release of norepinephrine we initially tested whether these agents could directly stimulate neurite outgrowth using primary neurons in culture [13], [36]–[38]. Fig. 4 shows that incubation of rat primary cortical neurons with either amphetamine (AMPH 10 uM) or norepinephrine (NE 10 uM) for 24 hr had no effect on neurite growth.

### Neurite outgrowth in primary cultured neurons is stimulated by factors secreted from astrocytes exposed to adrenergic agonists

**Figure 5:**
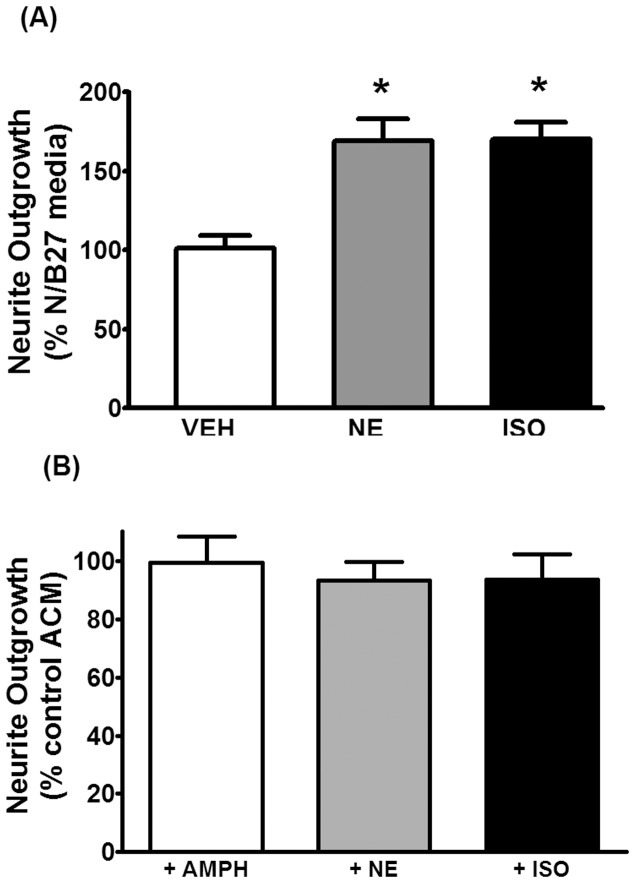
Conditioned media from astrocytes stimulated by norepinephrine or isoproterenol enhance neurite outgrowth in primary cortical neurons. (A) Conditioned media was obtained by incubating primary astrocytes, derived from cortices of postnatal day 2 rat pups as described in Methods, in N/B27 media containing vehicle, norepinephrine (NE; 10 uM) or the beta adrenergic agonist isoproterenol (ISO; 1 uM) for 6 hr. The conditioned media was collected, concentrated 10-fold by ultrafiltration through a 7000 MW cutoff membrane and then was applied to primary cortical neurons seeded on poly-lysine coverslips in N/B27. Following 24 hr incubation neurons on coverslips were fixed and stained for MAP2 and analyzed for neurite outgrowth as described in Methods. Data represent the mean ± SEM of 5 independent observations. (B) Conditioned media was obtained by incubating primary astrocytes, derived from cortices of postnatal day 2 rat pups as described in Methods, in N/B27 containing vehicle only for 6 hr. Following concentration by ultrafiltration the control conditioned media along with an aliquot of freshly prepared amphetamine (10 uM final concentration), NE (10 uM final concentration) or ISO (1 uM final concentration) were applied to primary cortical neurons seeded on poly-lysine coverslips in N/B27. Following 24 hr incubation neurons on coverslips were fixed and stained for MAP2 and analyzed for neurite outgrowth as described in Methods. Data represent the mean ± SEM of 4 independent observations.

Noradrenergic stimulation has been shown to upregulate FGF-2 expression in astrocytes [39]. To ascertain if this represented a mechanism underlying stimulated neurite outgrowth we carried out further investigations in vitro. Primary astrocytes from postnatal day 2 rat cortex were incubated for 6 hr in N/B27 with or without the addition of adrenergic agents. The conditioned media obtained was then incubated for 24 hr with primary cortical neurons from embryonic day 18 rat cortex and neurite growth was assessed. Fig. 5-A demonstrates that neurons incubated in conditioned media from astrocytes exposed to norepinephrine (10 uM) or isoproterenol (beta adrenergic agonist; 1 uM) for 6 hr exhibited a significant increase (approximately 70%) in neurite outgrowth as compared to neurons incubated in unconditioned N/B27 or conditioned media from astrocytes exposed to vehicle. Fig. 5-B demonstrates that co-incubation of neurons with conditioned media from unstimulated astrocytes (i.e. not exposed to adrenergic agents) along with direct addition of either norepinephrine (10 uM), isoproterenol (1 uM) or amphetamine (10 uM) led to no stimulatory effect on neurite outgrowth. Taken together these data indicate that noradrenergic stimulation of astrocytes, and not just coincident stimulation of astrocytic factors and noradrenergic agents, is a prerequisite to the neurite-promoting properties of astrocyte conditioned media.

### Pharmacology of adrenergic receptor subtypes mediating secretion of neuritogenesis-promoting factors suggest beta adrenergic and alpha1 adrenergic receptors are involved

**Figure 6:**
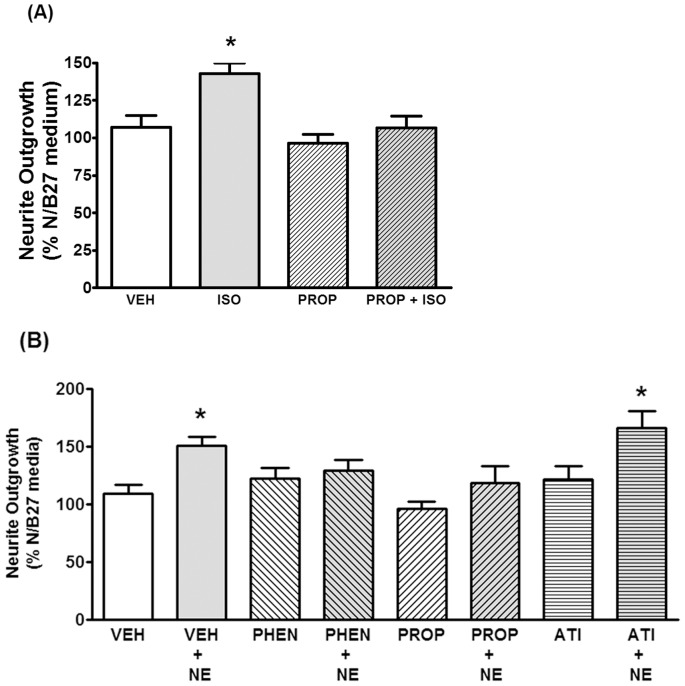
Both alpha- and beta-adrenergic antagonists prevent adrenergic agonists from stimulating astrocytes to produce neurite-promoting factors. (A) Astrocytes were incubated in N/B27 media to which vehicle (VEH), isoproterenol alone (ISO; 1 uM), the beta antagonist propranolol alone (PROP; 1 uM), or isoproterenol plus propranolol (ISO+PROP) were added just prior to 6 hr incubation. Following concentration by ultrafiltration the conditioned media were applied to primary cortical neurons seeded on poly-lysine coverslips in N/B27. Following 24 hr incubation neurons on coverslips were fixed and stained for MAP2 and analyzed for neurite outgrowth as described in Methods. Data were analyzed by one-way ANOVA followed by Bonferroni multiple comparisons of selected groups (i.e. VEH vs VEH+ISO, PROP vs PROP+ISO). Data represent the mean ± SEM of 5 independent observations. *, P<0.05 as compared to VEH. (B) Astrocytes were incubated in N/B27 media to which vehicle (VEH) or the indicated drugs were added just prior to 6 hr incubation. VEH = vehicle, NE = norepinephrine (10 uM), PHEN = phentolamine (1 uM), PROP = propranolol (1 uM), ATI = atipamezole (1 uM). Following concentration by ultrafiltration the conditioned media were applied to primary cortical neurons seeded on poly-lysine coverslips in N/B27. Following 24 hr incubation neurons on coverslips were fixed and stained for MAP2 and analyzed for neurite outgrowth as described in Methods. Data were analyzed by one-way ANOVA followed by Bonferroni multiple comparisons of selected groups (i.e. VEH vs VEH+NE, PHEN vs PHEN+NE, PROP vs PROP+NE, ATI vs ATI+NE). Data represent the mean ± SEM of 5 independent observations. *, P<0.05.

Inclusion of the beta adrenergic antagonist propranolol (PROP) during the 6 hr incubation of isoproterenol with astrocytes completely blocked the neurite-promoting effects of isoproterenol-conditioned medium when subsequently applied to cortical neurons (Fig. 6-A). Interestingly, the neurite-promoting effects of norepinephrine-conditioned medium were attenuated when either propranolol alone or phentolamine alone (PHEN; an alpha adrenergic antagonist) were included with norepinephrine during incubation with astrocytes (Fig. 6-B). By contrast, the alpha2 antagonist atipamezole (ATI) had no effect when co-incubated with norepinephrine during exposure to astrocytes. These data indicate that beta adrenergic and alpha1-, but not alpha2-, adrenergic stimulation of astrocytes can lead to the production of secreted astrocytic factors that promote neurite growth in neurons.

### Identification of FGF-2 as an essential component of neurite-promoting astrocyte conditioned media

**Figure 7:**
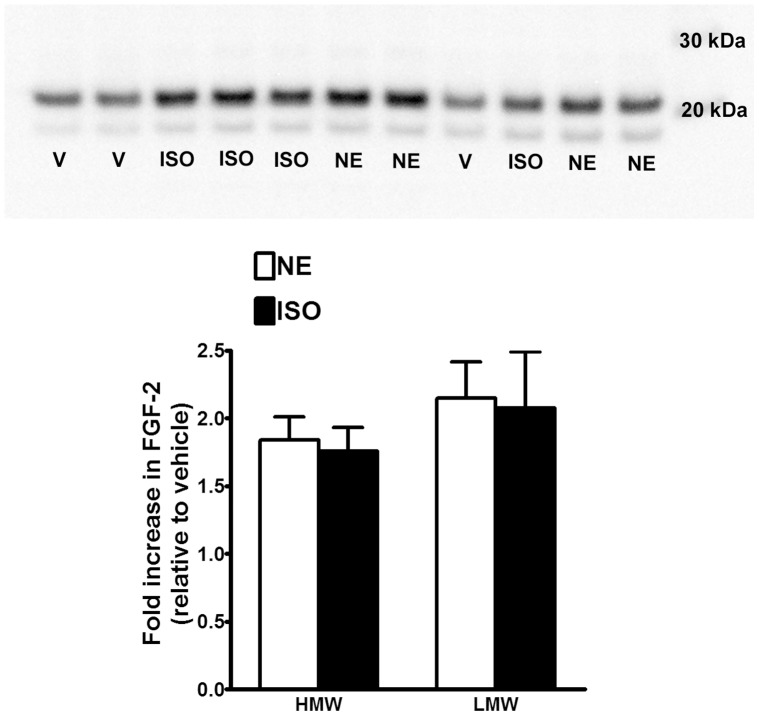
Norepinephrine and isoproterenol stimulate the production of high and low molecular weight forms of FGF-2 in astrocytes. Astrocytes were incubated in N/B27 media to which vehicle (V), norepinephrine alone (NE; 10 uM) or isoproterenol alone (ISO; 1 uM) were added just prior to 6 hr incubation. After 6 hr, astrocytes were harvested and cell lysates run on 10% reducing SDS-PAGE followed by immunoblotting for FGF-2 as described in Methods. FGF-immunoreactive bands ran at an approximate molecular weight of 23 kDa and 18 kDa, which are referred to as high molecular weight (HMW) and low molecular weight (LMW) isoforms, respectively. Data represent the mean ± SEM of 6 independent observations.

**Figure 8:**
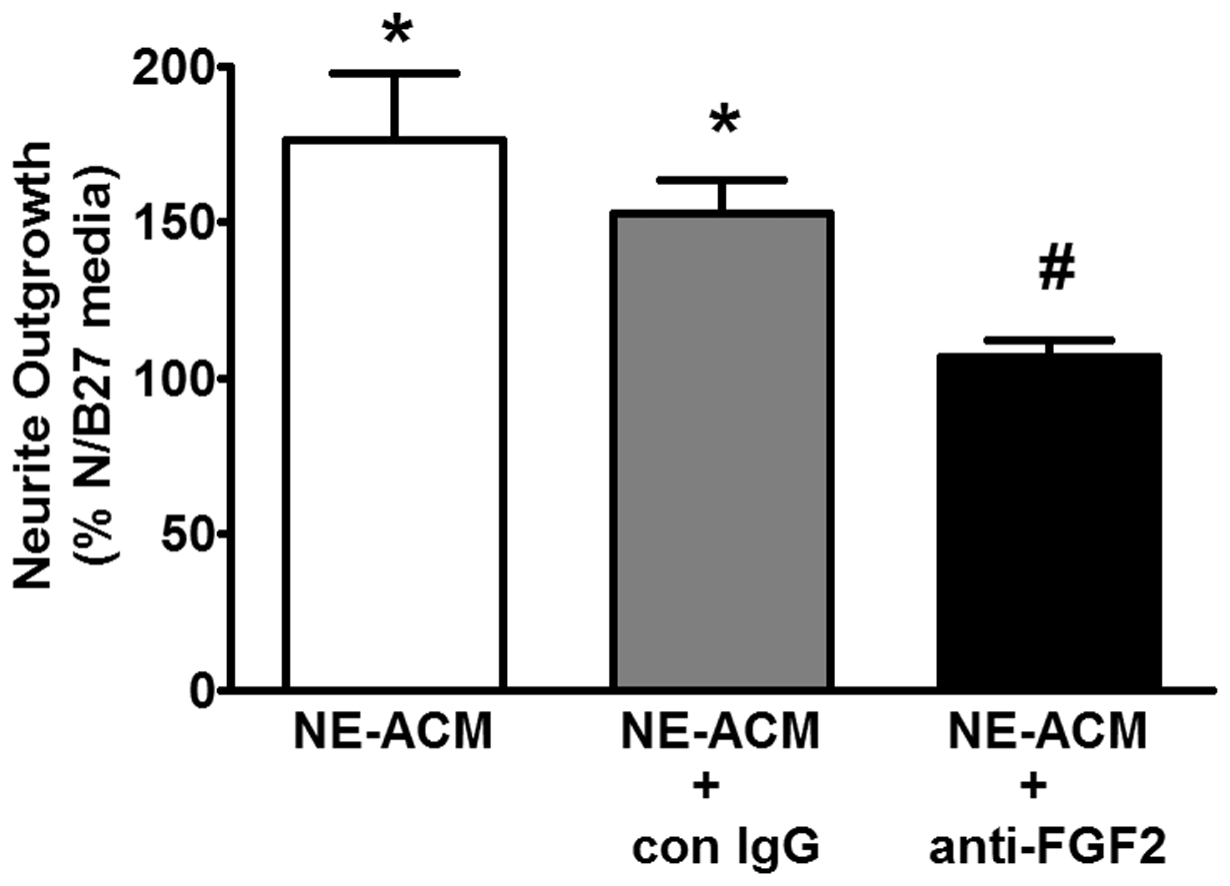
Neutralizing antibody to FGF-2 blocks neurite-promoting effects of astrocyte conditioned media from noradrenergically stimulated astrocytes. Astrocytes were incubated in N/B27 media to which norepinephrine (NE; 10 uM) was added just prior to 6 hr incubation. After 6 hr, conditioned media (NE-ACM) was collected and concentrated 10-fold by ultrafiltration through a 7000 MW cutoff membrane. The conditioned media was incubated for 30 min at room temperature in the presence of anti-FGF-2 neutralizing antibody (2.5 ug/ml goat polyclonal from R&D systems) or control IgG (2.5 ug/ml goat IgG) prior to applying to neuronal cultures on coverslips. Following 24 hr incubation neurons on coverslips were fixed and stained for MAP2 and analyzed for neurite outgrowth as described in Methods. Data were analyzed by one-way ANOVA followed by Student-Newman-Keuls comparisons. Data represent the mean ± SEM of 3 independent observations. *, P<0.05 as compared to unconditioned N/B27 media; #, P<0.05 as compared to NE-ACM or NE-ACM+con IgG.

To establish that FGF-2 contributes to the neuritogenesis caused by conditioned media from adrenergically-stimulated astrocytes we first demonstrated that NE or isoproterenol upregulated FGF-2 expression in astrocytes. Fig. 7 demonstrates that 6-hr exposure of astrocytes to drugs increased the expression of FGF-2. This figure depicts an increase of close to 2-fold in expression of both high and low molecular weight forms of FGF-2 in astrocyte lysates. Next, we took conditioned media from NE-stimulated astrocytes and pre-incubated with either a neutralizing FGF-2 antibody or control IgG prior to applying the media to neuronal cultures. Fig. 8 demonstrates that the neutralizing antibody significantly attenuated neurite outgrowth induced by the NE-stimulated astrocyte conditioned media. These data demonstrate that FGF-2 is an essential component of the neurite-promoting effects of conditioned media from noradrenergically stimulated astrocytes.

## Discussion

The present work extends our previous observations on pharmacological adjuncts in stroke rehabilitation. In this study we provide evidence to support FGF-2 as a mechanism underlying the neural plasticity and neurorestorative properties of amphetamine in stroke. We found that, following stroke in rats, short-term amphetamine paired with physical therapy induced an increase in the number of FGF-2 expressing pyramidal neurons in the unlesioned motor cortex as compared to physical therapy alone (Fig. 3). This effect was evident at two weeks post-stroke (approximately one week after cessation of amphetamine), but not at 8 weeks suggesting a relatively short-lived event. Nevertheless, at 8 weeks post-stroke animals given amphetamine paired with physical therapy had significantly greater axonal outgrowth in corticorubral pathways that originated from these layer V pyramidal neurons in the unlesioned motor cortex (Fig. 2-C). This neural plasticity was associated with significantly improved skilled motor function over physical therapy alone (Fig. 1-A,B). In fact, a significant correlation between midline fiber crossing in the corticorubral pathway and motor performance was observed (Fig. 2-D,E). In our mechanistic studies we found that noradrenergic stimulation of astrocytes can led to the secretion of FGF-2 which, in turn, could stimulate neurite outgrowth in primary cortical neurons. Taken together these data suggest a scenario in stroke rehabilitation in which amphetamine-induced norepinephrine release can trigger the upregulation of FGF-2 which could contribute to the neural plasticity observed.

In the stereological analysis of FGF-2 expression pyramidal cells in the unlesioned cortex were identified by their distinct morphology and clear evidence of staining. Although no formal attempt was made to assess the intensity of cellular staining it appeared that FGF-2-like immunoreactivity in animals given amphetamine was more intense than in animals that had received vehicle. Previous studies have shown amphetamine to modulate FGF-2 expression. In these studies short-term amphetamine (3 injections, once every other day) induced a persistent increase in FGF-2 in the brain that lasted for at least 1 month [30], [31]. In addition central administration of a neutralizing antibody to FGF-2 blocked the persistent plasticity-related behavioral/motor changes (referred to as “sensitization”) caused by amphetamine [31]. Interestingly, these authors observed the increase in FGF-2 to be attributed largely to astrocytes with effects being dependent on dosing and duration of amphetamine treatment [33]. The fact that we observed an increase in neuronal FGF-2-like immunoreactivity may relate to differences in treatment paradigms (e.g. amphetamine dosing or time point of analysis), immunohistochemical procedures and/or in the brain regions analyzed (cortex vs. midbrain). It seems reasonable to suggest that the increase in neuronal FGF-2-like immunoreactivity reflects either an upregulation in neuronal expression of FGF-2 or the sequestration of FGF-2 that has been released from neighboring cells, such as astrocytes. Another interesting point is that despite the observation that FGF-2 expression was a relatively short-lasting phenomenon (i.e. observable at week 2 but not week 8) axonal growth from the unlesioned pyramidal corticorubral projection neurons at study endpoint (week 8) was observed. This may reflect our inability to detect subtle differences in FGF-2 expression by immunohistochemistry or the possibility that a transient elevation in FGF-2 can act as a trigger to engage mechanisms of lasting neural plasticity and that protracted elevation in FGF-2 is not required.

To directly study a potential role for FGF-2 and the involvement of astrocytes in mediating neurite outgrowth we turned to a cell culture model. Since a major pharmacological action of amphetamine is to induce the release of norepinephrine we tested the effects of amphetamine and adrenergic agents on neurite outgrowth in rat cortical primary neurons in culture. Our results demonstrated that there was no direct effect of drugs on neurite outgrowth (Fig. 4), but clearly indicated that adrenergic stimulation of astrocytes leads to the secretion of factors that, when exposed to primary cortical neurons, promoted neurite outgrowth (Fig. 5-A). Coincident stimulation of neurons with adrenergic agents carried in from conditioned media was unlikely to be responsible as co-incubation of neurons with astrocyte conditioned medium from unstimulated astrocytes plus exogenously added drugs had no effect on neurite outgrowth (Fig. 5-B). Identification of FGF-2 as a critical neurite-promoting factor was obtained through the use of an FGF-2 neutralizing antibody, which when co-incubated with astrocyte-conditioned media prior to exposure to neurons, prevented neurite outgrowth (Fig. 8). These data are consistent with literature describing the neurite-promoting properties of FGF-2 [40]–[42].

A pharmacological analysis of the adrenergic receptors involved in stimulating astrocyte production of neuritogenic factors revealed that both alpha and beta subtypes were involved. Conditioned media from astrocytes exposed to the non-selective beta agonist, isoproterenol, induced an equivalent amount of neurite outgrowth as conditioned media from norepinephrine-stimulated astrocyes (Fig. 5-A). Conditioned media in which the beta adrenergic antagonist propranolol was present during incubation with isoproterenol had no neuritogenic effect on cortical neurons indicating that isoproterenol was acting through beta receptor stimulation (Fig. 6-A). Consistent with this finding are results from a recent study that demonstrated the ability of beta2-selective receptor agonists to stimulate astrocytes to produce neurite-promoting conditioned media [43]. By comparison, in the present study we found that when norepinephrine was the agonist either the alpha-adrenergic antagonist phentolamine or the beta-adrenergic antagonist propranolol was able to attenuate the ability of norepinephrine to induce astrocytes to secrete neurite-promoting factors (Fig. 6-B). These data suggest the involvement of alpha receptor subtypes. However, when the alpha2-selective antagonist atipamezole was tested it had no effect when co-incubated with norepinephrine during exposure to astrocytes. Thus, it appears that in addition to beta-adrenergic receptors, alpha1-, but not alpha2-, receptors can lead to the production of secreted astrocytic factors that promote neurite growth. This latter point is important in a translational context as alpha2 receptors function as inhibitory autoreceptors in the brain and alpha2 antagonists could be used to enhance norepinephrine release that would stimulate astrocytes to secrete FGF-2 [44]. In fact, our lab has shown that atipamezole administration after stroke in rats leads to improved rehabilitation-aided motor recovery [20].

Consistent with the involvement of alpha1 receptors and the concept that FGF-2 may be a critical neuritogenic component of astrocyte conditioned media is the observation that alpha1-adrenergic stimulation of the FGF-2 promoter leads to an upregulation in FGF-2 synthesis [45]. Our findings that noradrenergic agonists increase FGF-2 synthesis in astrocytes paralleled our observations on the production of neurite-promoting conditioned media and complement a recent study reporting on the effects of noradrenergic drugs on FGF-2 gene expression in astrocytes [46]. Finally, our observation that FGF-2 neutralizing antibodies abrogated the neuritogenic properties of conditioned media from stimulated astrocytes lend further support for our hypothesis that the noradrenergic stimulation of astrocytes represents an important mechanism leading to neurite outgrowth in vivo. Along these lines a number of preclinical and clinical studies have demonstrated a hindering effect of alpha adrenergic antagonists on motor recovery following stroke and other forms of brain damage [47]–[49].

The potential clinical utility of FGF-2 has been recognized for years. More recently, an analysis of gene expression associated with the learning of skilled motor tasks shows the involvement of FGF-2-related genes in this process [50]. Given this information, it seems reasonable to suggest that FGF-2 plays a key role in the development of neural plasticity related to motor rehabilitation following brain damage, such as in stroke. In the past a great deal of research investigating a therapeutic role for FGF-2 following ischemic damage had focused on its ability to stimulate neurogenesis or act as a neuroprotective agent in acute stroke. Preclinical studies had demonstrated that application of FGF-2 before or within hours of stroke could reduce infarct size [51]–[55]. However, clinical trials to assess the therapeutic efficacy of FGF-2 in acute stroke patients were halted due to severe hypotension and other negative consequences raising the question of whether neuroprotection in acute stroke is the most appropriate clinical application for FGF-2 [56]–[58]. The potential for FGF-2 as a neurorestorative adjunct in stroke was first demonstrated in studies in which central administration of FGF-2 within 24 hr of MCAO induced improved motor recovery and the upregulation of growth-associated protein 43, a marker of axonal growth [27], [28]. Conversely, central application of neutralizing antibodies to FGF-2 impaired motor recovery following aspiration lesions of the cortex [29]. Despite such intriguing results there has been little follow-up investigation on a “neurorestorative” role for FGF-2 in rehabilitative therapy following stroke.

In a larger context our findings are consistent with the wealth of preclinical studies that show that noradrenergic activation enhances motor rehabilitation after brain damage [21], [48], [49], [59], [60]. Additionally, there are a number of preclinical studies indicating that antidepressants that increase noradrenergic activity in the brain upregulate FGF-2 [61], [62]. Finally, norepinephrine-activating antidepressants have shown some promise to enhance motor rehabilitation following stroke, although this may be the result of a remission of depression leading to improved efficacy of rehabilitation regimens rather than a direct consequence of noradrenergic activation [63]–[65].

Stroke remains a leading cause of disability worldwide for which the development of more effective rehabilitative strategies is needed. Amphetamine, which induces the neuronal release of catecholamines (norepinephrine, dopamine), has been one of the most widely studied drugs for improving motor function following stroke [13]–[19]. Amphetamine has shown great promise in pre-clinical studies, but has produced mixed resulted in clinical trials [14], [17], [19], [21], [22]. The variability in clinical efficacy combined with the negative cardiovascular side effects have led to the conclusion at this point that the benefits of amphetamine do not outweigh the risks [17], [22]. Nevertheless a greater understanding of the salient mechanisms underlying amphetamine-enhanced motor improvement following stroke would facilitate the development of safer, more effective therapies. To that end, our present studies suggest that FGF-2 may represent such a mechanism. Studies are currently underway to directly test the therapeutic potential of FGF-2 upregulation in motor rehabilitation following stroke.
